# Chronic kidney disease, immunosuppression and tuberculin test sensitivity

**DOI:** 10.4103/0971-4065.78088

**Published:** 2011

**Authors:** S. A. Zaki

**Affiliations:** Department of Pediatrics, Lokmanya Tilak Municipal General Hospital, Mumbai, India

Sir,

I read with interest the recent report on “Tuberculin skin test for the diagnosis of latent tuberculosis during renal replacement therapy in an endemic area: A single center study” by Agarwal *et al*.,[1] and have the following comments to offer.

Host resistance to infection is primarily mediated by cellular immunity which is deficient in patients with chronic kidney disease (CKD). The occurrence of infections including tuberculosis (TB) is therefore high in such patients. In addition, various studies have reported the incidence of TB in patients with CKD and on maintenance hemodialysis to be 6–16 times that of general populations.[1,2] Impaired cellular immunity suppresses the mitogenic response of lymphocytes. Protein malnutrition, zinc and pyridoxine deficiency, and defects in leukocyte function following exposure to dialysis membranes increase the susceptibility of dialysis patients to TB.[2] Reaction size limits for determining a positive tuberculin test result vary with the individual’s risk of infection. Those with highest risk of having infection progress to disease – either having diseases associated with immunosuppression or on immunosuppressive therapy – a reactive area ≥ 5 mm is classified as positive result.[3] The authors have considered induration of 10 mm or more as positive for the diagnosis of latent TB (LTB) in their study. I feel that if the cut-off for tuberculin positivity was taken as 5 mm, the number of cases diagnosed as LTB would have definitely increased.
